# Self-Assembly of a Dipeptide with a Reduced Amount of Copper into Antifungal and Antibacterial Particles

**DOI:** 10.1021/acs.biomac.3c01092

**Published:** 2024-01-22

**Authors:** Michaela Kaganovich, Mohammad Taha, Uri Zig, Edit Y. Tshuva, Deborah E. Shalev, Abraham Gamliel, Meital Reches

**Affiliations:** † Institute of Chemistry, The Hebrew University of Jerusalem, Jerusalem 9190401, Israel; ‡ The Center for Nanoscience and Nanotechnology, The Hebrew University of Jerusalem, Jerusalem 9190401, Israel; § Hevel Maon Enterprises, Negev 8551900, Israel; ∥ Wolfson Centre for Applied Structural Biology, The Hebrew University of Jerusalem, Jerusalem 9190500, Israel; ⊥ Department of Pharmaceutical Engineering, Azrieli College of Engineering, Jerusalem 9103501, Israel; # Laboratory for Pest Management Research, Institute of Agricultural Engineering, ARO—The Volcani Center, Rishon LeZion 7505001, Israel

## Abstract

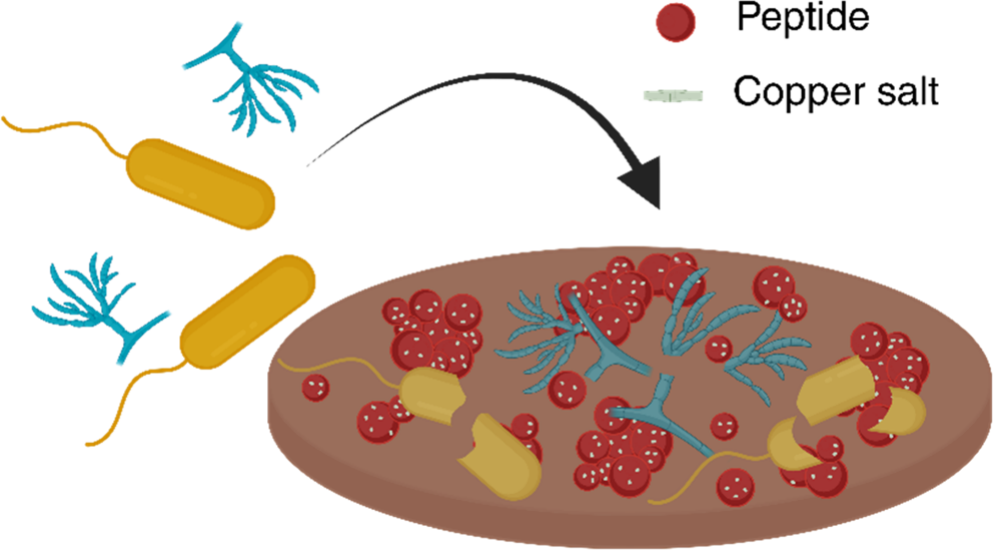

With the growing concern over the environmental impact and health risks associated with conventional pesticides, there is a great need for developing safer and more sustainable alternatives. This study demonstrates the self-assembly of antimicrobial and antifungal spherical particles by a dipeptide utilizing a reduced amount of copper salt compared to the commonly employed formulation. The particles can be sprayed on a surface and form an antimicrobial coating. The effectiveness of the coating against the bacteria *Pectobacterium brasiliense*, a common pathogen affecting potato crops, was demonstrated, as the coating reduced the bacterial load by 7.3 log. Moreover, a comprehensive field trial was conducted, where the formulation was applied to potato seeds. Remarkably, it exhibited good efficacy against three prevalent potato pathogens (*P. brasiliense*, *Pythium* spp., and *Spongospora subterranea*) while demonstrating no phytotoxic effects on the potatoes. These findings highlight the tremendous potential of this formulation as a nonphytotoxic alternative to replace hazardous pesticides currently available in the market.

## Introduction

Crop pathogens are a serious concern in the field of agriculture as they cause a threat to the productivity and quality of crop yields. These harmful microorganisms, such as bacteria, viruses, and fungi, can cause devastating diseases in various crops, leading to substantial economic losses and food security issues.^1^ Notably, the most consumed crops—wheat, maize, potato, rice, and soybean—face losses ranging from 17 to 30% due to pathogens and pests.^2^

Potato crop, being one of the most extensively cultivated crops, is highly susceptible to a range of pathogens that can result in significant loss of harvest.^1,3^ Among the common pathogens that pose a severe threat to potatoes are *Pectobacterium brasiliense*, *Pythium* spp., and *Spongospora subterranea*. *P. brasiliense* is the primary pathogenic factor contributing to the global prevalence of soft rot and blackleg diseases in potato tubers and stems.^4^ These bacteria are known to produce large quantities of pectic enzymes, which break down pectin in plant cell walls, leading to cellular damage and collapse. *P. brasiliense* bacteria are present on plant surfaces and in soil, and they can enter plants through wounds or natural openings like lenticels.^5^ They remain in these locations until the environmental conditions—such as the presence of free water, oxygen availability, and appropriate temperature—become favorable to the development of diseases.^5^ In addition to bacterial pathogens, fungi are commonly found in soil and water. One prevalent pathogenic fungus found in such an environment is *Pythium* spp. Once *Pythium* spp. infects a potato, it causes a condition known as “damping-off” in seedlings, where the young plants collapse and die before or shortly after emergence. In mature plants, *Pythium* spp. can cause root rot, leading to stunted growth, wilting, and eventual death.^6^ Another damaging pathogen is *S. subterranea*, which causes a significant disease known as a powdery scab in various root and tuber crops, most notably potatoes. It is a soil-borne pathogen that affects underground plant parts, primarily the tubers. Infection by *S. subterranea* leads to the formation of characteristic scab-like lesions on the surface of the tubers, resulting in a rough and pitted appearance.^7,8^

These diseases pose a substantial economic threat to agriculture, leading to decreased yields and compromised crop quality. To effectively combat these harmful pathogens, the utilization of pesticides becomes essential. However, the use of pesticides presents a high risk to both the environment and human health. These chemical substances are extensively used in agricultural practices worldwide. While pesticides have undoubtedly contributed to increased crop yields and improved food production, their extensive usage and accumulation in the environment have raised concerns regarding their adverse effects. The harmful impacts of pesticides include not only the intended targets but also nontargeted organisms, soil and water ecosystems, and the overall balance of the environment. Additionally, pesticide residues can reach the food chain, potentially posing a risk to human health.^9,10^

Therefore, there is an urgent need to develop environmentally friendly formulations with antimicrobial properties specifically designed for agricultural applications.^11^ Currently, the application of high dosages of copper-based pesticides, such as Mastercop, is common. Copper-based materials are the most widely utilized pesticides due to their numerous benefits and the absence of viable alternatives. Copper possesses several advantages, including its broad range of effectiveness against various pathogens, its ability to maintain high efficacy even in rainy conditions, its multisite mechanism of action that minimizes the emergence of resistant strains, its relatively low toxicity, and its low cost. Despite these advantages, the accumulation of copper in soil leads to prolonged environmental contamination, resulting in both bioaccumulation and toxicity. Hence, reducing the usage of copper-based plant-protection products is a top priority in the agriculture field. Consequently, it is crucial to minimize the quantity of copper applied.^11,12^

Peptides are of great promise as antimicrobial agents in agriculture due to their exceptional biocompatibility, low toxicity, and wide-ranging efficacy.^13−16^ Additionally, specific peptides possess the ability to efficiently bind to metal,^17−20^ potentially leading to a reduction in metal levels within the pesticide formulation.

We have demonstrated the ability of short peptides to self-assemble into spherical structures, exhibiting antiviral activity against bacteriophage T4 and Canine coronavirus.^21,22^ The peptide comprises two amino acids: l-3,4-dihydroxyphenylalanine (DOPA), which facilitates the attachment of the peptide to various surfaces, and phenylalanine, which enhances the self-assembly of the peptide through π–π interactions. The antiviral activity of the peptide assemblies is attributed to their interaction with the T4 phage’s neck protein. Through this interaction, the peptide assemblies destroy the viral protein structure, leading to an antiviral effect. The peptide DOPA-Phe-NH_2_ displayed an antiviral minimal inhibitory concentration of 125 μg/mL. When applied on the surface through drop-casting, it effectively deactivated over 99.9% of bacteriophage T4 and Canine coronavirus.

Here, we show that the combination of this dipeptide with a minimal amount of copper salt results in a remarkably effective antimicrobial and antifungal agent for crop protection. Through the catechol group of DOPA, the peptide forms a complex with copper.^23,24^ Importantly, this complex shows no phytotoxic effects while effectively protecting the potato seeds. These significant findings provide a solid basis for the practical implementation of this technology in agriculture.

## Materials and Methods

### Materials

DOPA-Phe-NH_2_ was synthesized with a purity of higher than 95%. Copper chloride (99.9%) was purchased from Acros Organics. Sodium chloride and sodium acetate were purchased from Bio-Lab (Jerusalem, Israel). Acetic acid, deuterium oxide, 3-(4,5-dimethylthiazolyl)-2,5-diphenyltetrazolium bromide (MTT), and isopropanol were purchased from Sigma-Aldrich. Triple distilled water (TDW) was obtained by filtering distilled water through a Milli-Q water system (Millipore). LIVE/DEAD Bacterial Viability Kit (L13152) was purchased from Thermo Fischer Scientific. Difco LB broth and Difco Nutrient Agar were obtained from Becton Dickinson (New Jersey). *P. brasiliense* was kindly provided by Prof. Ester Segal. Potato seed tubers were supplied by the farm cooperative of the Maon region. Mastercop was purchased from ADAMA (Israel). Silicon wafers with a diameter of 10 cm were diced into 1 × 1 cm^2^ pieces before use (7100 2 in. Pro-Vectus, ADT). Titanium grids (pure carbon film 400 mesh) were purchased from Ted Pella Inc. (Redding, CA). Human ovarian A2780 cancer cells were purchased from the European Collection of Authenticated Cell Cultures. Fetal bovine serum, penicillin–streptomycin, l-glutamine, and Roswell Park Memorial Institute (RPMI) 1640 medium were obtained from Biological Industries (Beit HaEmek, Israel).

### Preparation of the Peptide Coating

The peptide DOPA-Phe-NH_2_, dissolved in TDW at a concentration of 300 mM, was added to a solution of CuCl_2_ at a concentration of either 1.5 mM Cu^2+^ or 15 mM Cu^2+^, resulting in a final peptide concentration of 1.5 mM. This solution was shaken at 150 rpm at room temperature for 0.5 h. Subsequently, silicon wafers that underwent O_2_ plasma treatment were immersed in the peptide solution as well as mixtures of peptide and copper and only copper overnight. Following the coating process, the silicon surfaces were thoroughly rinsed to ensure the complete removal of any unattached peptide or copper salt. They were immersed in a TDW volume of 150 mL for a total of 10 times. Then, the rinsed silicon surfaces were placed in a vial containing 1 mL of TDW and subjected to shaking for a duration of 1 h. Finally, the coated surfaces were dried using nitrogen gas.

### Scanning Electron Microscopy (SEM) and Energy-Dispersive X-ray Spectroscopy (EDS)

The morphology and elemental composition of the samples were analyzed using an analytical high-resolution SEM Apreo 2S (Thermo Fisher Scientific) equipped with an EDS detector consisting of an UltraDry Premium 60 mm^2^ Silicon Drift detector (SSD) operating under the Pathfinder platform. SEM images were acquired under specific conditions, including an acceleration voltage of 2 kV, a current of 0.1 nA, and a working distance of 4 mm. EDS analysis was performed at an operating voltage of 5 kV, a current of 1.6 nA, and a working distance of 4 mm. The images were analyzed using ImageJ software.

### High-Angle Annular Dark-Field Scanning Transmission Electron Microscopy (HAADF-STEM)

The titanium grids underwent a 30 s treatment with O_2_ plasma, following which a 3 μL sample drop was placed on them and dried under ambient conditions. The grids were subsequently examined using an analytical high-resolution SEM Apreo 2S (Thermo Fisher Scientific) equipped with a high-angle annular dark-field scanning transmission electron microscopy (HAADF-STEM) detector. The analysis was done at an operating voltage of 25 kv, a current of 1.6 Na, and a working distance of 10 mm.

### Atomic Force Microscopy (AFM)

The surface roughness and topography of the silicone surfaces were assessed using AFM. AFM measurements were performed with JPK NanoWizard3 instrument (JPK, Berlin, Germany) operating in tapping mode. For the measurements, a tip with a spring constant of 3 N/m and a resonant frequency of 75 kHz was used (Aspire, Team Nanotec GmbH, Villingen-Schwenningen, Germany).

### Transmission Electron Microscopy (TEM)

The morphology of the peptide assemblies was characterized using TEM. A drop of 5 μL of a peptide solution at a concentration of 300 mM was applied to the carbon–Formvar coated copper grids 200 mesh for 40 s, and the excess solution was blotted with a filter paper. The sample was analyzed using a Tecnai 12 TEM 120 kV instrument (Phillips, Eindhoven, The Netherlands) equipped with a Phurona camera and RADIUS software (Emsis GmbH, Münster, Germany).

### Circular Dichroism (CD)

CD spectra were obtained using a J-810 spectropolarimeter (JASCO, Tokyo, Japan) equipped with a 0.1 cm path length quartz cuvette for far-UV CD spectroscopy. Measurements were conducted at a temperature of 20 °C within the spectral range of 190–260 nm, with a step width of 0.05 nm. The samples were dissolved in TDW to achieve a final peptide concentration of 0.3 mM and subsequently filtered using a 0.22 μm filter. Three spectra were collected, averaged, and background-subtracted for each sample by using TDW as the reference.

### Fourier Transform Infrared Spectroscopy (FT-IR)

A peptide solution at a concentration of 1.5 mM, a 1:1 mixture of the peptide and Cu^2+^ solution, and a 1:10 mixture of the peptide and Cu^2+^ solution were applied onto CaF_2_ plates and subjected to vacuum drying. Subsequently, the peptide and mixtures on the plates were resuspended in D_2_O and subjected to vacuum drying. This was repeated twice to eliminate the hydration layer consisting of H_2_O from the plate. To obtain FT-IR spectra, a Nicolet 6700 FT-IR spectrometer equipped with a deuterated triglycine sulfate (DTGS) detector (Thermo Fisher Scientific, MA) was used. Transmittance measurements were conducted over the wavenumber range of 400–4000 cm^–1^ with a resolution of 4 cm^–1^. To ensure accurate data acquisition, the spectra were averaged after 600 scans.

### Fluorescence Measurement

A peptide dissolved in TDW at a concentration of 900 mM was added to a solution of CuCl_2_ at a concentration of either 4.5 mM Cu^2+^ or 2.2 mM Cu^2+^, resulting in a final peptide concentration of 4.5 mM. In addition, separate solutions consisting of only peptide and only copper were prepared using the same procedure. The solutions were shaken at 150 rpm at room temperature for 0.5 h. Emission measurements were conducted using a spectrofluorometer (Jasco FP8200ST, Japan). The excitation wavelength was set at 370 nm, with a slit width of 10 nm, and emission signals were recorded within the range of 420–600 nm. The slit width for emission was maintained at 2.5 nm.

### Absorption Spectroscopy

The solutions were prepared following the exact procedure described in the Fluorescence Measurement section. The absorbance of the solutions was then measured by using a UV/vis spectrophotometer (Shimadzu, UV-1650PC, Kyoto, Japan).

### Nuclear Magnetic Resonance (NMR)

NMR samples were prepared using 4.0 mM peptide in 20 mM acetate buffer at pH 5.0 with 10% deuterium oxide. NMR experiments were performed at 20 °C with a Bruker AVII 500 MHz spectrometer operating at a proton frequency of 500.13 MHz, using a 5 mm selective probe with a self-shielded xyz-gradient coil. Correlation spectroscopy (COSY)^25^ and rotating frame Overhauser effect spectroscopy (ROESY) experiments using an optimized mixing time of 400 ms^26,27^ were performed using gradients for water suppression under identical conditions.

Spectra were processed and assigned using the TopSpin (Bruker Analytische Messtechnik GmbH) and NMRFAM SPARKY software.^28^

### Laser Confocal Scanning Microscope (CLSM)

CLSM images were obtained by using an FV-1200 confocal microscope (Olympus, Japan) equipped with a 60*X*/1.42 oil immersion objective. Green fluorescence was visualized by using a 488 nm excitation filter and a 500–540 nm emission filter.

### Antibacterial Activity

The antibacterial activity was evaluated using *P. brasiliense*. A starter culture of the bacteria was prepared by adding a single colony to 10 mL of LB solution and incubating it overnight at 28 °C at 150 rpm. The bacterial suspension was subsequently centrifuged at 4000 rpm for 3 min and washed three times using phosphate-buffered saline (PBS) buffer (10 mM, pH 7.0, 154 mM NaCl). The bacterial suspension was diluted to 10^6^ (CFU/mL) by measuring the optical density (OD) at 600 nm. A 15 μL drop of *P. brasiliense* suspension, at a concentration of 10^6^ CFU/mL, was placed on each surface (1 cm × 1 cm). The surfaces were then incubated at 28 °C for 24 h. Afterward, they were sonicated in 3 mL of PBS buffer for 1 min, vortexed, and decimally diluted to 10^–3^. The bacterial suspension at different dilutions was seeded on solidified nutrient agar and incubated at 28 °C for 24 h. Finally, the colonies were counted, and the number of CFU per mL was calculated based on the number of colonies, dilution factor, and the volume of the bacterial suspension. To ensure the reliability of the results, the antibacterial activity assay was repeated three times in three independent experiments, resulting in a total of nine replicates for each surface.

### Membrane Integrity Assessment (Live/Dead Assay)

The viability of *P. brasiliense* cells was determined by using a live/dead assay. *P. brasiliense* suspension, with a concentration of 1 × 10^6^ CFU/mL, was placed on the silicon surfaces at 28 °C for 24 h, as described above. Subsequently, the surfaces were sonicated in 1 mL of PBS buffer for 1 min and vortexed. The resulting solution was then mixed with a stain mixture consisting of propidium iodide (75 μM) and SYTO 9 (15 μM). Then, the stained bacteria solution was incubated for 15 min in the dark at room temperature. To visualize the stained bacterial cells, a 5 μL drop of the stained solution was placed on the microscope glass slide and covered with cover glass. The images of the stained bacterial cells were observed using CLSM, with excitation wavelengths of 488 and 561 nm.

### Potato Seeds Tuber Coating

Peptide with copper suspension and a commercial copper pesticide, Mastercop, diluted 6.7 times according to the manufacturer’s specifications, were applied to potato seed tubers (Sifra, size of 45–60 mm diameter) in a specially designed commercial aerosol chamber. Potato tubers were surface brushed and passed on a roller conveyor through a spray chamber. The spray chamber consists of pneumatic nozzles generating a mist cloud of sprayed suspensions (median diameter of droplets 50 μm). The tubers were exposed to the aerosol for 30 s and coated with a dense droplet coverage (1500–2000 droplets/cm^2^). The coated tubers were packed and shipped for planting.

### Field Experiment

The coated and uncoated potato seed tubers were planted in the soil of a commercial potato field of the farm cooperative of the Maon region. The crop was grown for 120 days, after which the tubers were harvested. The harvested potatoes were visually inspected for the presence of three specific pathogens: *P. brasiliense*, *Pythium* spp., and *S. subterranea*.

### Cell Culture and Cell Viability Measurements

Cytotoxicity was measured on human ovarian A2780 cancer cells through the MTT assay.^29^ The cells were cultured in RPMI 1640 medium supplemented with 10% fetal bovine serum, 1% penicillin–streptomycin, and 1% l-glutamine. Then, the cells were seeded at a density of 0.6 × 10^6^ cells per well in a 96-well plate and allowed to attach for 1 day at 37 °C in a 5% CO_2_ atmosphere in RPMI 1640 medium. The test samples, dissolved in TDW at a 21 mM concentration, underwent serial dilution to create a concentration gradient using pure TDW as the control. The resulting solutions were added to the cells, with the highest concentration set at 2.1 mM. Following a three-day incubation period at 37 °C in a 5% CO_2_ atmosphere, MTT (0.1 mg in 20 μL) was added, and the cells were further incubated for an additional 3 h. Subsequently, the MTT solution was removed, and 200 μL of isopropanol was added. The absorbance at 550 nm was measured using a Spark 10 M multimode microplate reader spectrophotometer (Tecan Group Ltd.). Each measurement was repeated at least three times per plate, all repeated at least three times on different days, resulting in a minimum of nine repetitions. The relative IC_50_ values were determined by a nonlinear regression of a variable slope (four parameters) model by using GraphPad Prism 5.04 software and reported as mean ± standard deviation (SD).

## Results and Discussion

### Preparation of the Peptide Coating

The peptide concentration in the formulation was optimized using three different peptide concentrations: 30, 6, and 4 mM. SEM analysis showed that surfaces coated with the peptide at different concentrations, as well as surfaces coated with peptide in combination with the highest concentration of Cu^2+^ (30 mM), exhibited the formation of a substantial and dense layer (Figure S1a,b,c,e). Lower peptide concentrations (6 mM and 4 mM) with Cu^2+^ led to the formation of spherical structures embedded within a thick peptide coating (Figure S1d,f). To improve the uniformity of the coating, we decreased the peptide concentration to 1.5 mM.

**Scheme 1:**
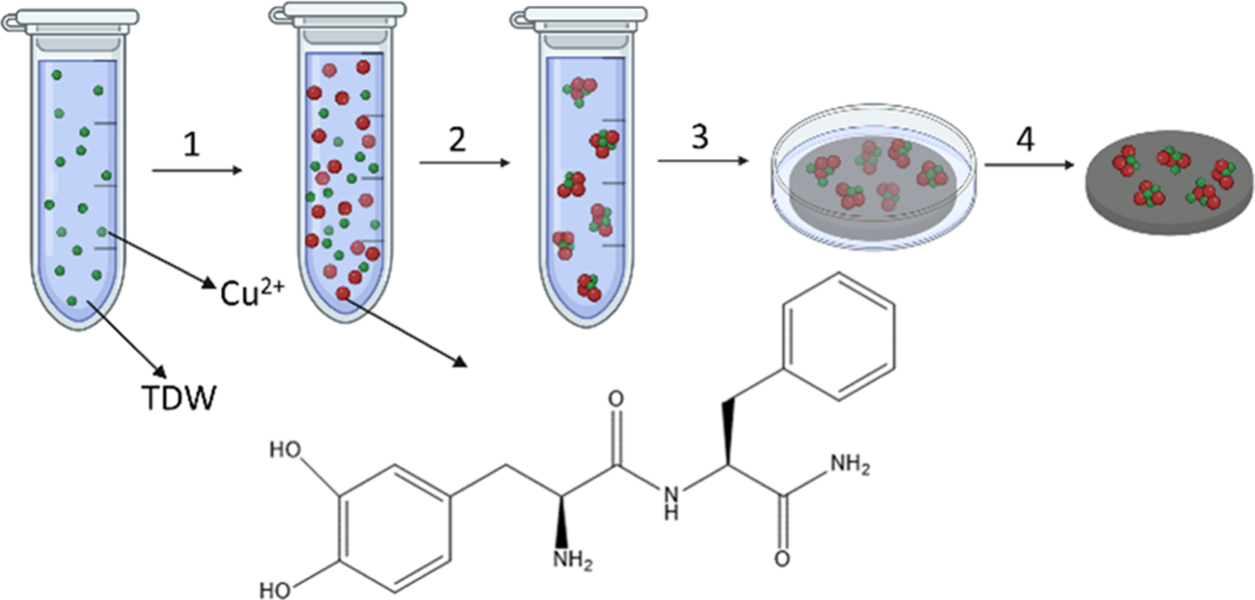
Schematic Illustration of the Coating Preparation First, a solution of Cu^2+^ (represented by green circles) was prepared (step 1). Then, the peptide (represented by red circles) was added to the solution of Cu^2+^ (step 2). The silicone surface (represented by a gray surface) was immersed in this mixture overnight (step 3). After the immersion period, the surface was extracted, rinsed by TDW, and dried with nitrogen (step 4).

To determine the effect of different copper concentrations on peptide self-assembly, the peptide (1.5 mM) was mixed with one equivalent or with 10 equiv of Cu^2+^. We termed these mixtures PepCu1 and PepCu10, respectively. We first applied these mixtures on flat silicon surfaces by immersing them overnight in a solution that contained either the dipeptide or the dipeptide together with copper (PepCu1 or PepCu10). In addition, a copper salt solution was applied as a coating to serve as the control sample. Subsequently, the surfaces were thoroughly washed with TDW to ensure the removal of unattached peptide or copper salt (Scheme 1).

### Characterization of the Peptide Assemblies

**Figure 1:**
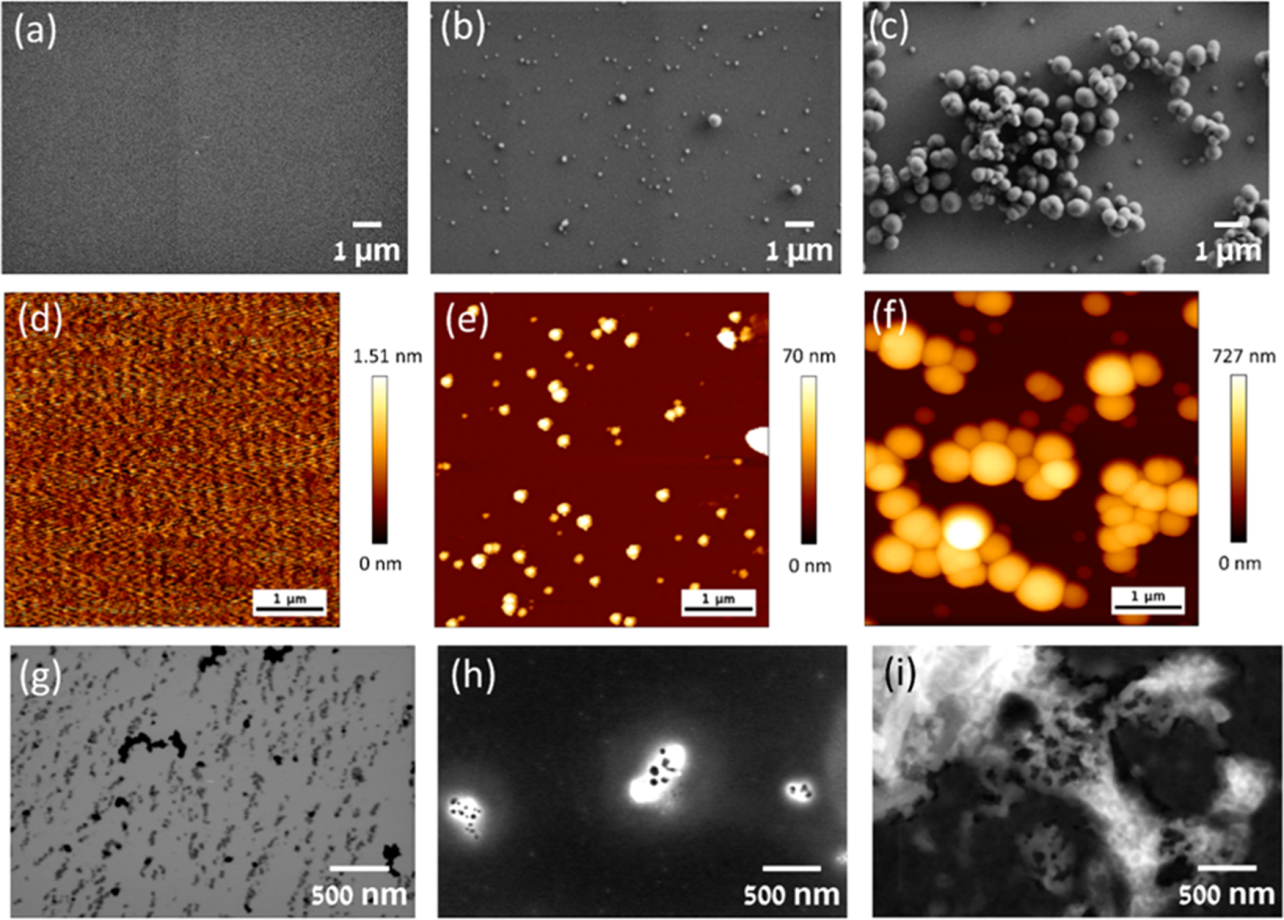
Characterization of the coating. (a, d, and g) SEM, AFM, and HAADF-STEM images of the peptide; (b–h) SEM, AFM, and HAADF-STEM images of PepCu1; (c–i) SEM, AFM, and HAADF-STEM images of PepCu10. Images (a–f) were taken after washing the coated surfaces with TDW.

To examine the resulting assemblies on the surfaces, scanning electron microscopy (SEM) and atomic force microscopy (AFM) were employed for analysis. Silicone-coated surfaces, prepared as outlined in Scheme 1, were used for both SEM and AFM analyses. The peptide mixtures, PepCu1 and PepCu10, exhibited the formation of spherical structures on the silicon surface (Figure 1b,c,e,f). Notably, PepCu1 showed structures with a diameter of 60 ± 30 nm, while PepCu10 displayed larger structures with a diameter of 275 ± 100 nm. In contrast, no structures or aggregates were detected for the peptide solution without copper salt (Figure 1a,d). These results are in accordance with high-angle annular dark-field scanning transmission electron microscopy (HAADF-STEM) analysis where relatively small peptide structures could be detected for the peptide solution (Figure 1g), while spherical structures could be detected for both PepCu1 and PepCu10. The observed particles were dark, surrounded by white regions (Figure 1h,i). This noticeable image contrast in HAADF-STEM analysis indicates the presence of a heavy element, presumably copper, which appears brighter, while the lighter element, carbon, appears darker. Additionally, TEM analysis revealed that at a peptide concentration of 300 mM (Figure S3), only small aggregates are formed, similar in morphology to those observed without copper at a peptide concentration of 1.5 mM. Interestingly, the presence of copper at a concentration of 1.5 mM results in larger particles displaying defined spherical structures. The observed increase in particle size and the emergence of well-defined structures suggest that the presence of copper plays a crucial role in influencing the self-assembly process, leading to the formation of larger and more organized peptide–copper complex structures.

**Figure 2:**
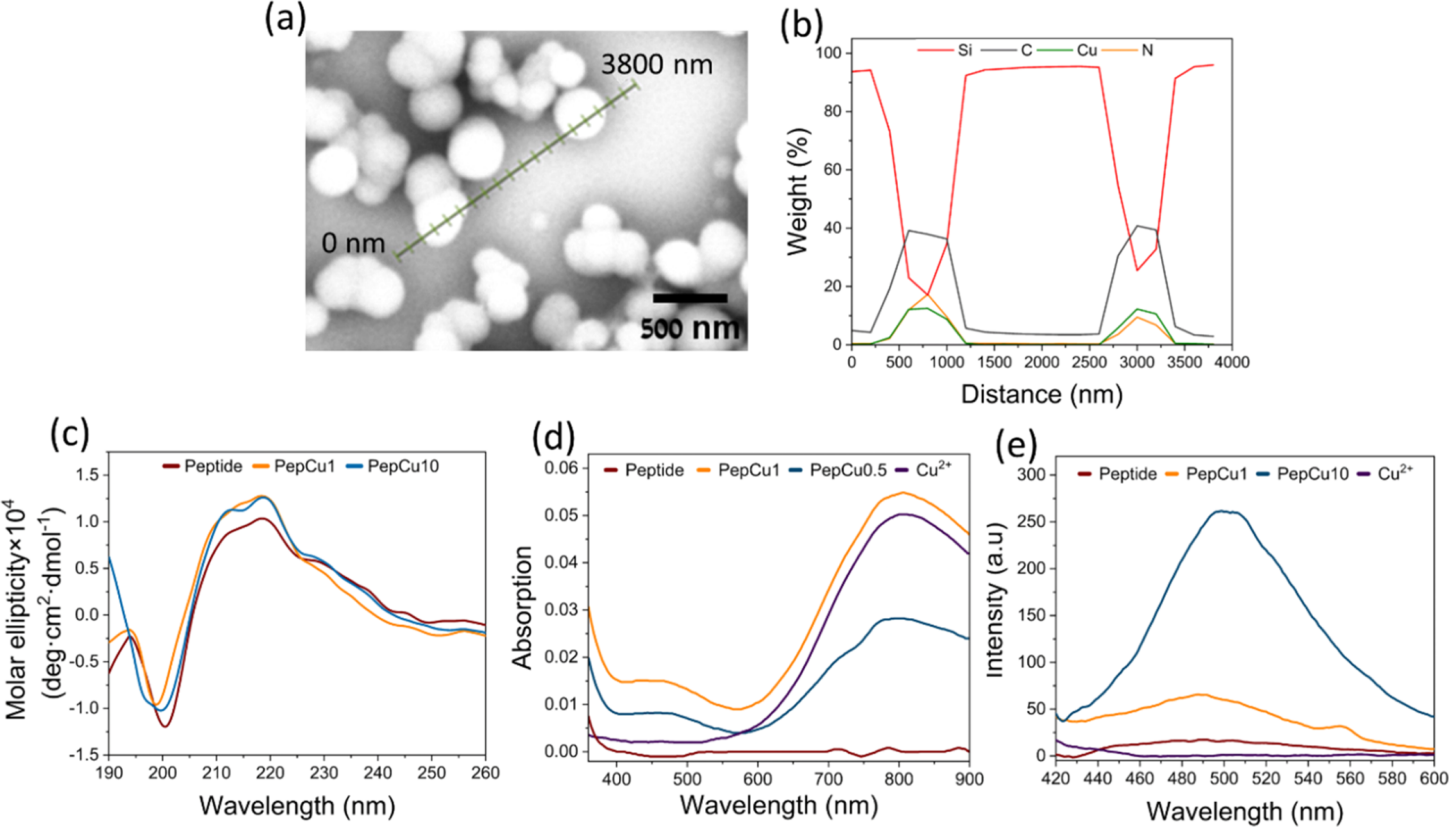
Elemental composition of the coating and characterization of the peptide assemblies in the solution. (a and b) SEM and EDX line scan analysis of the PepCu10 surface, (c) CD spectra for the peptide, PepCu1, and PepCu10, (d) absorbance spectra for the peptide, copper, PepCu1, and PepCu0.5, and (e) emission spectra of Peptide, PepCu1, PepCu10, and Cu^2+^ at an excitation of 370 nm.

Energy-dispersive X-ray spectroscopy (EDX) line scan analysis was conducted on a line passing through two particles of the PepCu10 sample, as observed in the SEM micrograph (Figure 2a). The line scan was performed at 20 different positions along the line (Figure 2a). The EDX analysis revealed an increase in the elemental composition of carbon, copper, and nitrogen within the two particles, indicating the presence of both peptide and copper. Conversely, in other areas examined, these elements were not detected (Figure 2b).

Circular dichroism (CD) analysis was conducted to evaluate the secondary structure of the peptide (Figure 2c). The CD spectra obtained for the peptide with and without copper displayed a negative peak around 200 nm and a positive peak around 215 nm. The presence of these characteristic peaks indicates a β-turn structure of the peptide with and without copper.^30^ Furthermore, Fourier Transform Infrared Spectroscopy (FT-IR) analysis confirmed the secondary structure of the peptide. A peak at 1670 cm^–1^ in the spectra of the peptide and the peptide complexes (PepCu1 and PepCu10) indicates a β-turn structure (Figure S4).^31^

The effect of Cu^2+^ present in the peptide solution on the polymerization of DOPA was investigated by measuring the emission of the peptide solution at an excitation of 370 nm. A peak at 500 nm was observed for PepCu1 and PepCu10, with the intensity being 5 times higher in the case of PepCu10 (Figure 2e). In a solution of peptide without copper, a slight increase was observed, while a solution containing only copper salt did not exhibit this peak. The increased emission in the presence of copper indicates DOPA polymerization.^32^ Copper ions initiate the oxidation of DOPA, generating reactive intermediates that actively contribute to the polymerization of DOPA. This polymerization process induces structural and property changes in the peptide, ultimately influencing its interaction with the surface.

The binding stoichiometry between the catechol group of the peptide and Cu^2+^ was determined by absorbance. Two solutions were prepared to establish whether the stoichiometry between the peptide and Cu^2+^ is indeed 1:1 or 1:2. PepCu1 was prepared with a 1:1 ratio between the peptide and Cu^2+^, and PepCu0.5 was prepared with a ratio of 1:0.5 between the peptide and Cu^2+^.

The absorbance spectra of PepCu1 and PepCu0.5 exhibited a distinct absorption peak at 450 nm (Figure 2d), indicating the formation of a peptide–Cu complex at a ratio of 1:1.^23^ The peak observed at 805 nm for PepCu1, PepCu0.5, and Cu^2+^ alone is attributed to the presence of free Cu^2+^.^23^ Notably, the peptide solution did not exhibit any peak in this wavelength range.

**Figure 3:**
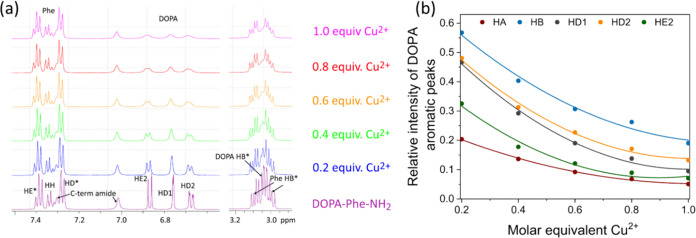
NMR characterization. (a) Aromatic region of the ^1^H NMR spectrum of titration of DOPA-Phe-NH_2_ peptide with Cu^2+^ shows an increased PRE, especially at the DOPA region of the peptide, as a function of increasing Cu^2+^ concentration, the inset shows the β-hydrogen region and (b) PRE reduction of NMR signal during titration of DOPA-Phe-NH_2_ peptide with Cu^2+^ for the hydrogen signals of the DOPA residue.

The NMR titration of the peptide with Cu^2+^ showed Cu^2+^ binding, evidenced by the paramagnetic relaxation enhancement (PRE) of the aromatic hydrogens that was significantly stronger in the DOPA hydrogen region than in the Phe region.^33^ The relative rate of reduction of the signal was measured as a function of the concentration of Cu^2+^ to indicate the mechanism of binding (Figure 3b). The signals showed little change in chemical shift as a function of titration, indicating that the free species are in slow exchange (*K*_d_ < 0.5 μM) with the bound species that is not evident due to broadening.^34^ The coefficient of the fitted relative reduction in signal as a function of titration with Cu^2+^ indicated the order of binding as being Hδ1 > Hβ > Hδ2 > Hε2 ≫ Hα. The chemical shifts of the peptide were assigned: DOPA Hα 4.06, Hβ 2.94, Hδ1 6.67, Hδ2 6.58, Hε2 6.77; Phe HN 8.36, Hα 4.49, Hβ 2.30, 2.91, Hδ 7.19, Hε 7.29, Hζ 7.25, C-terminus amide 7.27, 6.97.

### Antimicrobial Activity of the Peptide Assemblies

**Figure 4:**
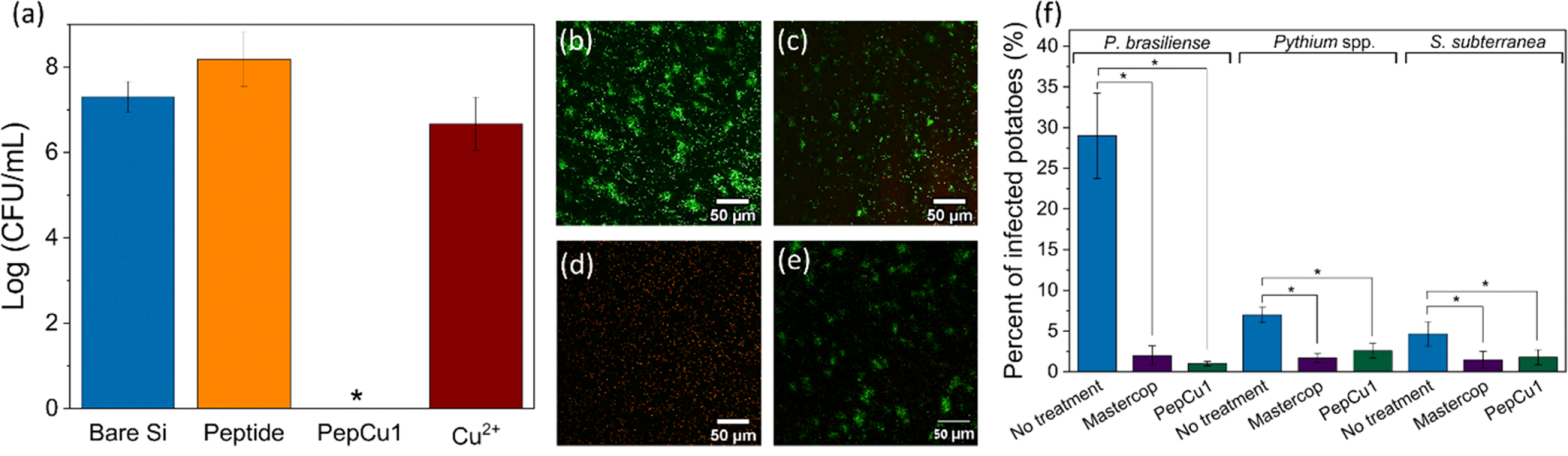
Antibacterial assays against *P. brasiliense* for the coated surfaces. (a) Number of colonies on four different surfaces: bare silicon, peptide-coated, PepCu1-coated, and coated with copper salt. The standard deviation (SD) was calculated using data from three independent experiments, each with three replicates, for a total sample size of nine. Live/dead assay microscopy images of *P. brasiliense* cells after incubation with four different surfaces: (b) bare silicon, (c) peptide coating, (d) PepCu1 coating, and (e) copper salt coating. Green-stained cells indicate live cells, while red-stained cells indicate dead cells. (f) Field experiment results of the percentage of infected potatoes by three pathogens: *P. brasiliense*, *Pythium* spp., and *S. subterranea* under different treatment conditions: without treatment, after treatment with a commercial product, Mastercop (130 mM Cu^2+^), and after treatment with PepCu1 (1.50 mM Cu^2+^). The standard deviation (SD) was calculated using data from one independent experiment, each with three or four replicates. Analysis of variance (ANOVA) test with Tukey–Kramer post hoc analysis was used to indicate statistically significant results. Statistical significance was determined at *p* < 0.05 and marked with one asterisk (*).

The antibacterial activity of the coated surfaces was measured after incubating the surfaces overnight with 10^6^ colony-forming units (CFU) per mL of bacterial strain *P. brasiliense*. After this incubation period, the bacteria that adhered to the surfaces were detached by sonication and collected. The collected bacterial solution underwent decimal dilution, and the diluted solution was subsequently plated on agar plates. After incubation for 24 h, the resulting colonies on the agar plates were counted and quantified. Surfaces coated with peptide or copper exhibited a high number of bacteria, 8.0 ± 0.4 log (CFU/mL) and 6.7 ± 0.6 log (CFU/mL), respectively. However, the PepCu1 complex coating demonstrated a significant reduction of 7.3 log (CFU/mL) compared to the noncoated surfaces (Figure 4a).

A live/dead assay provided additional verification of the antibacterial properties of the copper–peptide complex. The surfaces were incubated for 24 h in a suspension of *P. brasiliense*, followed by staining and visualization using confocal laser scanning microscopy (CLSM). The findings strongly support the high efficacy of PepCu1 against *P. brasiliense*, as evidenced by the presence of dead bacterial cells (displaying a red color) in comparison to the other surfaces: peptide-coated, bare silicon, and copper salt-coated (Figure 4b–e).

To evaluate the efficacy and potential applicability of PepCu1 in the agricultural domain, coated and uncoated potato seed tubers were tested. The potato seed tubers were coated by spraying the PepCu1 suspension in a designated aerosol chamber. A commercial copper pesticide, Mastercop, was used as a reference at the label concentration of 130 mM Cu^2+^. The concentration of copper in the Mastercop product was 89 times higher than that of the PepCu1 treatment. Additionally, a set of potato seed tubers served as untreated controls. Following coating, the potato tubers were planted in soil in a commercial field and grown under common production practices for 120 days until harvest. The harvested potato tubers were examined for visual symptoms of infection caused by three specific pathogens: *P. brasiliense*, *Pythium* spp., and *S. subterranea*. Validation of the presence of the pathogen was carried out by isolating the pathogen from the infected tissue of samples of tubers.

Mastercop and PepCu1 treatments significantly reduced the percentage of the three pathogens (Figure 4f). For *P. brasiliense*, the percentage decreased from 29% to 2% for Mastercop and 1% for PepCu1. Similarly, for *Pythium* spp., the percentage decreased from 7% to 1% for Mastercop and 3% for PepCu1. For *S. subterranea*, the percentage decreased from 7 to 3% for both Mastercop and PepCu1. It is worth noting that PepCu1 contains copper at a concentration 89 times lower than that found in Mastercop. These results indicate the remarkable effectiveness of the PepCu1 treatment, as it demonstrates that copper can be used at significantly reduced concentrations, which is advantageous for environmental considerations and human health.

The antimicrobial effectiveness of the PepCu1 complex is attributed to the presence of Cu^2+^ ions, which play a diverse role in disrupting cell membranes, binding to crucial proteins in microbial cells, initiating the generation of reactive oxygen species, interacting with microbial DNA, and coordinating with diverse cellular molecules.^35^ Furthermore, the antimicrobial properties of the complex can be associated with the catechol moieties present in the DOPA amino acids. Our earlier research has demonstrated that peptide assemblies containing DOPA-Phe-Phe exhibit antimicrobial properties.^36^

Besides mitigating the pathogen presence, this study also examined the impact of the treatments on potato yield and potential cytotoxic effects. The application of the PepCu1 treatment resulted in a potato yield comparable to that of the control group, which received no treatment (Figure S5).

These findings strongly indicate that the PepCu1 treatment does not exhibit any cytotoxic effects on the potatoes, further highlighting its safety and compatibility with crop production.

The peptide, PepCu1, copper salt, and Mastercop were evaluated for their cytotoxicity against ovarian carcinoma (A2780) human cancer cell lines within the concentration range of 0.01–2.1 mM. Cytotoxicity was assessed through the MTT assay following a 72 h incubation of the cells with the different compounds. Remarkably, the peptide demonstrated no cytotoxicity toward A2780 cells (Figure S6), while samples containing the copper salt exhibited comparable IC_50_ values of 1.1 ± 0.2, 1.2 ± 0.3, and 1.6 ± 0.3 mM for PepCu1, copper salt, and Mastercop, respectively (Figure S6). PepCu1 was applied to potato seed tubers at a concentration of 1.5 mM, approaching its IC_50_, while Mastercop was applied at a notably higher concentration (130 mM of Cu^2+^). These results clearly show that PepCu1 is markedly less toxic than currently employed formulations.

## Conclusions

In this study, we investigated the formation of the complex of the DOPA-Phe-NH_2_ peptide with Cu^2+^. The incorporation of Cu^2+^ facilitated the self-assembly of the peptide into spherical structures by attachment to the catechol group. The resulting complex displays the capability to attach to surfaces and demonstrates significant potential in effectively combating plant pathogens. When the complex was applied to potato seeds, it exhibited a significant decrease in both plant bacteria and fungi. Importantly, toxicity assessments revealed that the complex did not exhibit any adverse effects on the potatoes, highlighting its safety for agricultural applications. The peptide–Cu complex holds great potential for widespread use in agriculture as an effective defense against diverse plant pathogens.

## Supporting Information Available

SEM images of peptide-coated surfaces at varied concentrations of the peptide; AFM topography of bare silicone surface; TEM images of the peptide; FT-IR spectra of peptide, PepCu1, and PepCu10; categorization of potatoes yield quality in relation to infections; and viability of A2780 cells based on the MTT assay (PDF)
