# Supplementary material for: Self-Assembly of a Dipeptide with a Reduced Amount of Copper into Antifungal and Antibacterial Particles
Source: Biomacromolecules. 2024 Jan 22;25(2):1018–26. doi: 10.1021/acs.biomac.3c01092 (PMC11184556; doi:10.1021/acs.biomac.3c01092)
Supplement: Supplementary file 1 — bm3c01092_si_001.pdf [file bm3c01092_si_001.pdf]

Supplementary Information

Self-Assembly of a Dipeptide with a Reduced Amount of Copper into Antifungal and Antibacterial Particles

*Michaela Kaganovich[a,b], Mohammad Taha[a], Uri Zig[c], Edit Y. Tshuva[a], Deborah E. Shalev[d,e], Abraham Gamliel[f] and Meital Reches[a,b]**

[a]Institute of Chemistry, The Hebrew University of Jerusalem, Jerusalem, 9190401, Israel

[b]The Center for Nanoscience and Nanotechnology, The Hebrew University of Jerusalem, Jerusalem, 9190401, Israel

[c]Hevel Maon Enterprises, Negev, Israel

[d]Wolfson Centre for Applied Structural Biology, The Hebrew University of Jerusalem, Israel

[e]Department of Pharmaceutical Engineering, Azrieli College of Engineering, Jerusalem, Israel

[f]Laboratory for Pest Management Research, Institute of Agricultural Engineering,

ARO – the Volcani Center, Rishon LeZion, Israel

*E-mail: meital.reches@mail.huji.ac.il

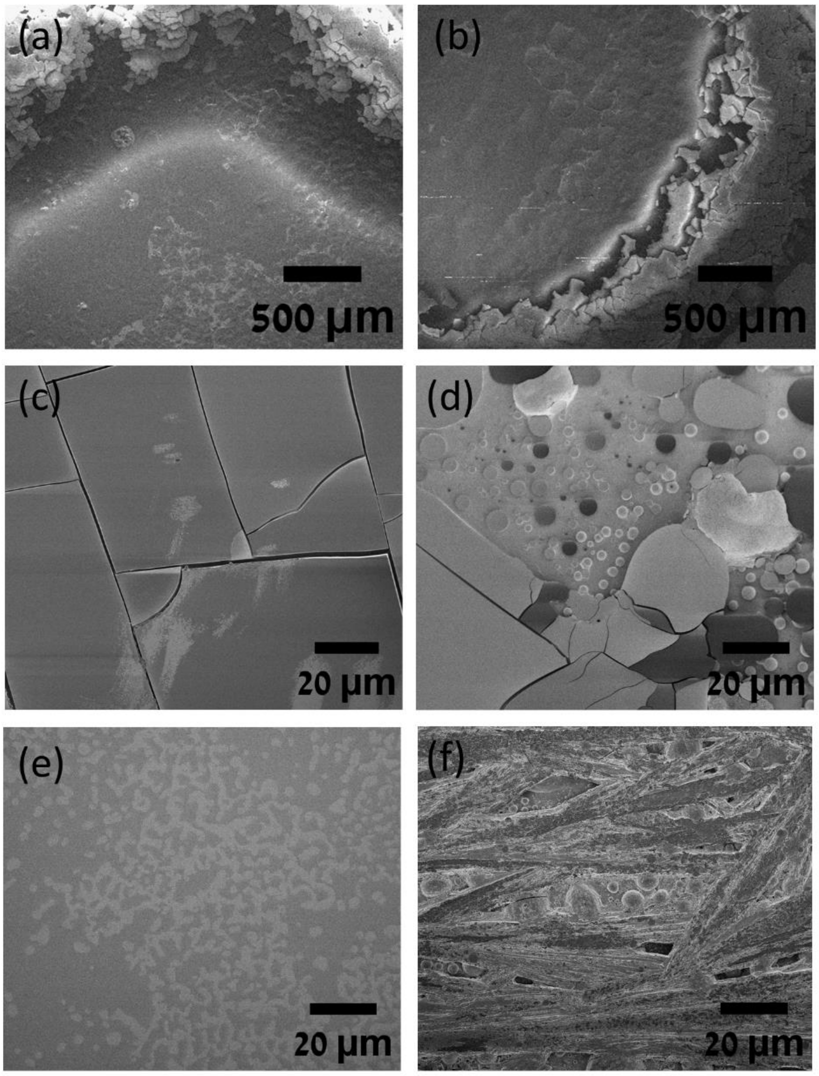

Figure S1. SEM images of surfaces. Peptide-coated surfaces at varied concentrations of the peptide: (a) 30 mM, (c) 6 mM, (e) 4 mM. Peptide-coated surfaces (b) 30 mM, (d) 6 mM, (f) 4 mM with copper at a concentration of 0.9 mM.

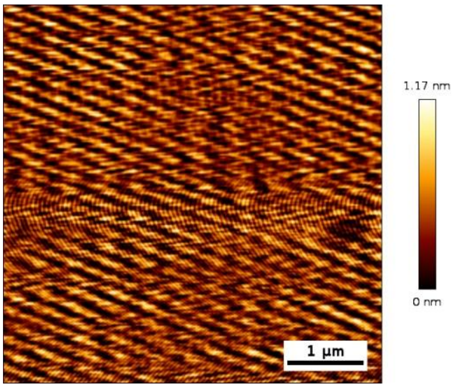

Figure S2. AFM topography of bare silicone surface.

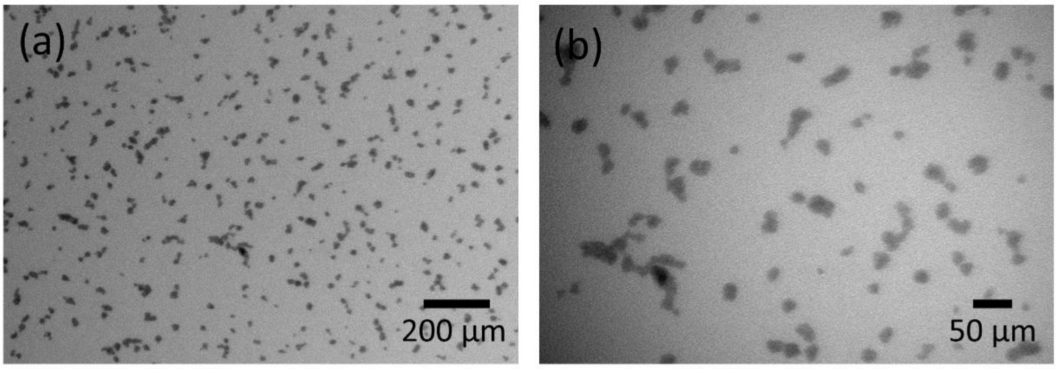

Figure S3. TEM images of the peptide at a concentration of 300 mM at different magnifications (a and b).

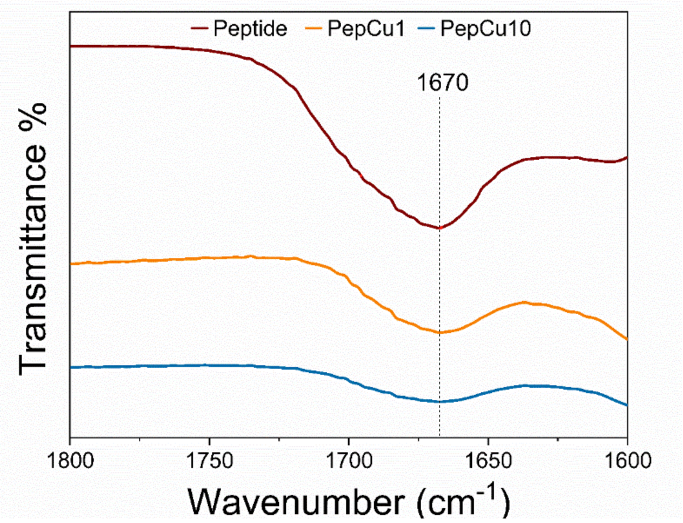

Figure S4. FT-IR spectra of peptide, PepCu1, and PepCu10.

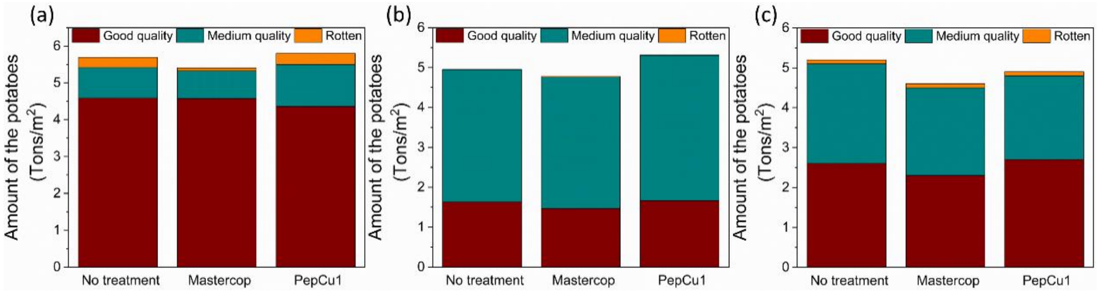

Figure S5. Categorization of potatoes yield quality in relation to *infections: (a) P. Brasiliense, (b) Pythium* spp., and *(c) S. subterranea* infection after no treatment, after

treatment with commercial product Mastercop® (114 mM Cu^{2+}), and after treatment with a PepCu1 (1.50 mM Cu^{2+}).

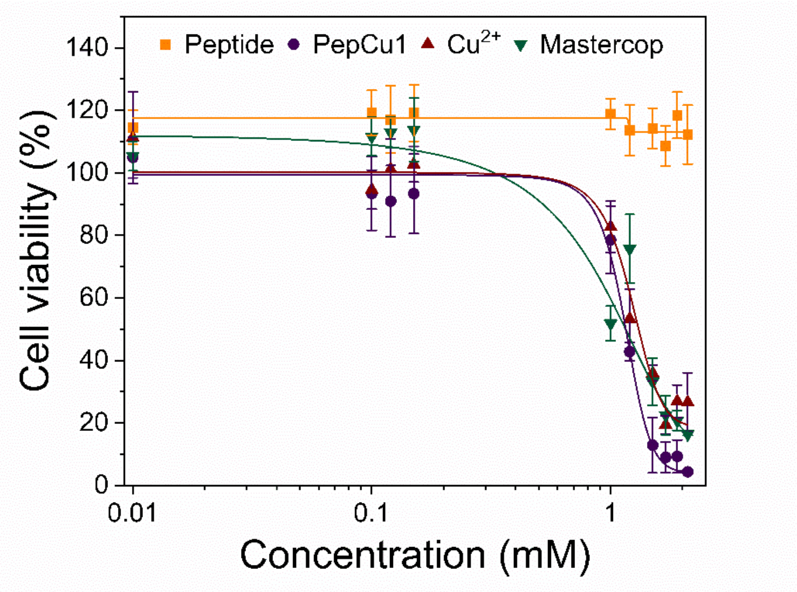

Figure S6. Viability of A2780 cells based on the MTT assay. Error bars indicate the standard deviations of data from three independent experiments, each with three replicates, for a total sample size of nine.
